# High-resolution structural variant profiling of myelodysplastic syndromes by optical genome mapping uncovers cryptic aberrations of prognostic and therapeutic significance

**DOI:** 10.1038/s41375-022-01652-8

**Published:** 2022-08-01

**Authors:** Hui Yang, Guillermo Garcia-Manero, Koji Sasaki, Guillermo Montalban-Bravo, Zhenya Tang, Yue Wei, Tapan Kadia, Kelly Chien, Diana Rush, Ha Nguyen, Awdesh Kalia, Manjunath Nimmakayalu, Carlos Bueso-Ramos, Hagop Kantarjian, L. Jeffrey Medeiros, Rajyalakshmi Luthra, Rashmi Kanagal-Shamanna

**Affiliations:** 1 Department of Leukemia, The University of Texas M.D. Anderson Cancer Center, Houston, TX USA; 2 Department of Hematopathology, The University of Texas M.D. Anderson Cancer Center, Houston, TX USA; 3 School of Health Professions, The University of Texas M.D. Anderson Cancer Center, Houston, TX USA

**Keywords:** Genetic testing, Myelodysplastic syndrome, Myelodysplastic syndrome, Disease-free survival, Translational research

## Abstract

Chromosome banding analysis (CBA) remains the standard-of-care for structural variant (SV) assessment in MDS. Optical genome mapping (OGM) is a novel, non-sequencing-based technique for high-resolution genome-wide SV profiling (SVP). We explored the clinical value of SVP by OGM in 101 consecutive, newly diagnosed MDS patients from a single-center, who underwent standard-of-care cytogenetic and targeted NGS studies. OGM detected 383 clinically significant, recurrent and novel SVs. Of these, 224 (51%) SVs, seen across 34% of patients, were cryptic by CBA (included rearrangements involving *MECOM, NUP98::PRRX2, KMT2A* partial tandem duplications among others). SVP decreased the proportion of normal karyotype by 16%, identified complex genomes (17%), chromothripsis (6%) and generated informative results in both patients with insufficient metaphases. Precise gene/exon-level mapping allowed assessment of clinically relevant biomarkers (*TP53* allele status, *KMT2A-PTD*) without additional testing. SV data was complementary to NGS. When applied in retrospect, OGM results changed the comprehensive cytogenetic scoring system (CCSS) and R-IPSS risk-groups in 21% and 17% patients respectively with an improved prediction of prognosis. By multivariate analysis, CCSS by OGM only (not CBA), *TP53* mutation and BM blasts independently predicted survival. This is the first and largest study reporting the value of combined SVP and NGS for MDS prognostication.

## Introduction

Myelodysplastic syndromes (MDS) are characterized by a high degree of somatic genetic alterations ranging from single-nucleotide mutations to intermediate and large-sized structural variants (SVs) [1–4]. SVs are defined as genomic alterations greater than 50 base pairs (bp) that encompass copy number variants (CNVs: deletions, and duplications), insertions, inversions, gene rearrangements, complex genomic alterations, and copy-neutral loss-of-heterozygosity (CN-LOH)/uniparental disomy [5, 6]. Identification of SVs, largely evaluated by conventional chromosome banding analysis (CBA), is critical for diagnosis, prognostic risk-stratification of MDS and therapeutic decision making [3, 4, 7–15]. Recent data in MDS, using *TP53* as a prototype, has highlighted the importance of assessment of both somatic mutations and SVs (deletions, CN-LOH, rearrangements) in genomic regions of interest for accurate prognostication [16, 17].

While the mutational landscape of MDS has been extensively characterized using next-generation sequencing (NGS), there is little information regarding unbiased high-resolution structural variant profiling (SVP) [2]. This is due to the limited ability of NGS to align and assemble segments from repetitive sequences, which constitute ~50% of human genome and harbor most of the complex SVs [18–21]. Therefore, to date, CBA remains the gold standard in the evaluation of MDS. However, CBA has limited resolution and poor sensitivity in detection of some clinically actionable alterations, and on occasions, can be non-informative due to the requirement of cell culture. In these contexts, and in the setting of failed or insufficient metaphases, as recommended by NCCN, these findings need to be supplemented with additional assay(s) such as fluorescence in situ hybridization (FISH) and chromosomal microarray (CMA), resulting in increasing costs and turn-around-times for the laboratory [22–24]. As a result, the knowledge of the full spectrum and prevalence of genome-wide high-resolution chromosomal SVs in MDS remains limited.

Optical genome mapping (OGM) is a novel, non-sequencing-based technique that enables detection of all SVs by evaluation of patterns generated by fluorophore tags labeled to specific 6 bp sequence motifs within extremely long DNA molecules (>300 kb), and is, therefore, unaffected by repeat sequences. The ability to detect all types of SVs in a single assay enabling unbiased structural variant profiling (SVP) at a high-resolution has been shown in constitutional and prenatal disorders, solid tumors, and a few hematological malignancies [25–30]. OGM lacks base-level resolution, which can be overcome by simultaneous targeted NGS-based mutational analysis, facilitating comprehensive evaluation of genomic landscape.

In this study, we performed pan-genomic profiling of a large cohort of newly diagnosed MDS patients and evaluated the impact on clinical prognostication using a combination of high-resolution SVP by OGM and mutation profiling by targeted deep NGS. The goal of this study was to identify the application of routine SVP in MDS by defining the spectrum of high-resolution SVs by OGM and evaluating the clinical impact of the information by clinicopathologic and outcome correlation.

## Materials and methods

### Sample selection

We used bone marrow (BM) aspirates from consecutive treatment naïve MDS patients who presented to our institution with adequate fresh/ frozen material available for OGM. The study was approved by the Institutional Review Board, all samples were collected following institutional guidelines with informed consent in accordance with the Declaration of Helsinki. Risk-stratification was performed using comprehensive cytogenetic scoring system (CCSS) and IPSS-R [3, 4].

### Cytogenetic and molecular testing

All patients underwent BM aspiration and biopsy for diagnosis per 2016 WHO criteria [7] and standard-of-care testing in CLIA-certified laboratories that included targeted 81-gene NGS panel (Supplementary Table S1) [31–36], CBA by traditional G-banding and FISH/CMA (as needed) per standard protocols described elsewhere [37–40]. Additional FISH or CMA testing was performed to confirm most of the clinically significant OGM findings that were cryptic by CBA. All details are in Supplementary Material.

### Optical genome mapping (OGM)

#### DNA extraction, labeling, and chip loading

We used BM mononuclear cells (BMMNCs) isolated from patient samples by ficoll density-gradient centrifugation that were either stored as viable cells in liquid nitrogen or cell pellets at −80 °C within 3 days of collection. Ultra-high molecular weight (UHMW) genomic DNA (gDNA) was extracted from 1.5 million cells following manufacturer’s protocols (Bionano Prep SP Blood and Cell DNA Isolation Kit; Bionano Genomics, San Diego, CA). Briefly, after thawing in 37 °C water bath, cryopreserved BMMNCs were washed with DNA stabilizing buffer to get the cell pellet, which was re-suspended in 40 ul DNA stabilizing buffer, lysed and digested with proteinase K, RNase A and buffer LBB. After PMSF treatment (Sigma-Aldrich, St. Louis, MO), nanobind paramagnetic disk was added to the solution, mixed with isopropanol to precipitate the gDNA and washed with WB1 and WB2. After transferring to a new tube, buffer EB (Bionano Genomics, San Diego, CA) was added to elute the gDNA from the disk. The gDNA was equilibrated overnight at room temperature for homogenization and subsequently quantified with Qubit Fluorometer (Qubit BR dsDNA assay kit; ThermoFisher Scientific, CA).

Sequence-specific Direct label and stain (DLS) technique was used for labeling following manufacturer’s protocols (Bionano Prep DLS Labeling Kit; Bionano Genomics, San Diego, CA). Direct Labeling Enzyme 1 (DLE-1) reaction was carried out using 750 ng of gDNA to tag a specific 6 bp sequence (CTTAAG) with a DL-green fluorophore (~15 times per 100 kb). Following puregene proteinase K (Qiagen, Hilden, Germany) digestion and DL-Green clean-up using DLS membrane in DLS 24-well plate, the labeled gDNA was mixed with DNA stain, stained overnight at room temperature for backbone visualization and quantified using Qubit HS dsDNA assay kit (ThermoFisher Scientific, CA). The fluorescent-labeled gDNA molecules were loaded on a Saphyr chip G2.3, and linear double stranded molecules passing across nanochannels were sequentially imaged on a Saphyr instrument (Bionano Genomics, San Diego, CA) (Supplementary Fig. S1).

### Data analysis and variant filtering

Effective genome coverage of approximately 300X was achieved for every tested sample (1300 GB data per sample), theoretically enabling detection of aberrations at a 5% allele frequency (equivalent to 10% of cells when heterozygous). Standard run quality control parameters [total DNA ( ≥150 kbp), N50 ( ≥150 kbp), map rate (≥150 kbp), effective coverage (>300x), average label density (per 100 kbp)] were evaluated per manufacturer’s guidelines. Details regarding limit of detection, reproducibility and precision are described in Supplementary Material [41, 42].

Data was analyzed using Bionano Access (Bionano Genomics, San Diego, CA) using Genome Reference Consortium GRCh38/hg38 as the reference. Data analysis was performed in a single-blinded fashion independently by 2 users using de novo (DN; SVs > 500 bp), rare variant (RV; SVs > 5000 bp) and copy number pipelines (CN; capturing large CNVs > 500,000 bp potentially missed by SV algorithms). RV pipeline enabled detection of SVs occurring at low allelic fractions (~10%), as determined by prior sensitivity studies using simulations and serial dilutions of cell lines (data not shown). DN pipeline was primarily used for CN-LOH assessment and confirmation of SV calls > 5000 bp detected by RV pipeline; SV calls between 500 bp and 5000 bp were not included for this study.

For variant filtering, first, we used the recommended size and confidence score filters for each of the three pipelines for to generate a list of high confidence SVs and copy number variants for analysis described elsewhere (Supplementary Table S2) [27, 28, 30, 41]. Second, we used the OGM data generated from 200 healthy individuals to select only rare variants that represent pathogenic somatic alterations. As a final third step, in order to select SVs that are of clinical significance, we selected SVs that overlapped the coding region of a gene/ chromosome locus implicated in myeloid neoplasm (Supplementary Table S3), adapted from the publicly available myeloid neoplasm-specific gene list [Cancer Genomics Consortium (https://www.cancergenomics.org/gene_lists.php)] and in-house 81-gene NGS panel. The final interpretation of every call was made after visualizing the changes in the sequence patterns in the molecules from sample compared to the reference.

### Statistical analysis

Overall survival (OS) was calculated from the time from diagnosis to death or the last follow-up date. Patients who were alive at their last follow-up were censored on that date. The Kaplan–Meier product limit method was used to estimate the median OS for each parameter. Univariate Cox proportional hazards regression analysis was used to identify association of each of the variables with OS, followed by multivariate analysis (using *p* = 0.2 cut-off to select variables). Prognostic fitness of cytogenetic risk calculated from CBA and OGM were assessed using Harrell’s concordance index.

## Results

### Baseline patient characteristics

The study cohort included 101 consecutive patients with newly diagnosed and treatment naïve MDS. Baseline patient characteristics are shown in Table 1. A total of 62 (62%) patients had at least 1 clonal cytogenetic alteration, with a median of 1 (0–12) abnormality per patient. Two patients had insufficient metaphases. The median number of somatic gene mutations was 2 (0–7) per patient. The most frequent mutations (seen in >5% of patients) were *TET2* (31%), *SF3B1* (28%), *TP53* (22%), *ASXL1* (17%), *RUNX1* (12%), *SRSF2* (9%), *DNMT3A* (7%), and *IDH1/2* (5%). The distribution of the cytogenetic risk by CCSS, R-IPPS risk categories and somatic mutations were representative of a real-world setting, with a slightly increased frequency of complex karyotype (CK, 29%) and/or *TP53* mutations (22%) due to tertiary care referral bias.

**Table 1:** Baseline characteristics of the MDS study cohort (*n* = 101).

Baseline characteristics (*n* = 101)	
Men	71 (71%)
Women	29 (29%)
Parameter	Median (range)
Age	72 (25–92) years
Complete blood counts	
Hemoglobin	9.2 (6.9–17.2)
Mean corpuscular volume	100 (77–122)
Platelet count	123 (9–660)
Absolute Neutrophil Count	1.5 (0–113)
Bone marrow	
Bone Marrow blasts%	3 (0–19)
Karyotype	
Normal karyotype	38 (38%)
Complex karyotype	29 (29%)
del(5q)/-5	26 (26%)
del(7q)/-7	16 (16%)
Abnormal chromosome 11	14 (14%)
Trisomy 8	12 (12%)
del(20q)	12 (12%)
Abnormal chromosome 12	11 (11%)
Abnormal chromosome 3	5 (5%)
Abnormal chromosome 17	5 (5%)
−Y	5 (5%)
Insufficient metaphases	2 (2%)
Number of somatic mutations	
0	15 (15%)
1	28 (28%)
2	24 (24%)
3	20 (20%)
>3	12 (12%)
Gene mutations	
* TET2*	31 (31%)
* SF3B1*	28 (28%)
* TP53*	22 (22%)
* ASXL1*	17 (17%)
* RUNX1*	12 (12%)
* SRSF2*	9 (9%)
* DNMT3A*	7 (7%)
Comprehensive cytogenetic score (CCSS)	
0 - Very Good	3 (3%)
1 - Good	43 (43%)
2 – Intermediate	22 (22%)
3 – Poor	10 (10%)
4 - Very Poor	21 (21%)
Not available	2 (2%)
R-IPSS risk categories	
Very low	5 (5%)
Low	26 (26%)
Intermediate	30 (30%)
High	16 (16%)
Very high	20 (20%)
Not available	2 (2%)
Treatment* (*n* = 99)	
Observation	63 (64%)
Disease modifying therapy	36 (36%)
A. HMA** single agent	13 (36%)
B. HMA with investigational agents***	18 (50%)
C. HMA with chemotherapy	2 (6%)
D. Chemotherapy only	3 (8%)
Allogeneic stem cell transplantation	12 (12%)

*Treatment during MDS phase available in 99 patients.

**HMA, hypomethylating agents included azacitidine, decitabine and guadecitabine.

***Investigational agents included the following: venetoclax, pembrolizumab, immune check point inhibitor, eprenetapopt (APR-246), IDH inhibitor, tyrosine kinase inhibitor, glutaminase inhibitor, AXL inhibitor.

### SVP of MDS using high-resolution OGM

**Fig. 1:**
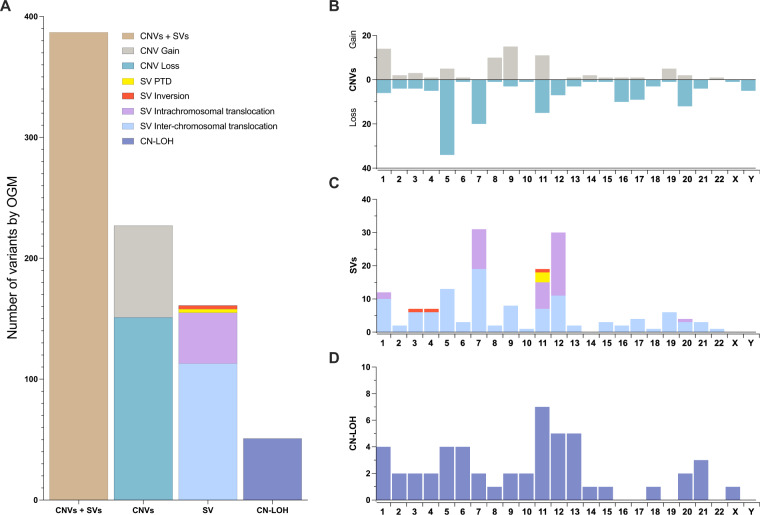
Overall distribution of frequencies of different types of clinically significant chromosomal aberrations in MDS detected by OGM represented by chromosome involvement. **A** A total of 383 of 4030 unique copy number variant (CNV) (both gains and losses) plus SV calls (with subtypes, included partial tandem duplications, all involving *KMT2A* on chromosome 11, inversions, intra-chromosomal and inter-chromosomal translocations), as well as copy neutral loss-of-heterozygosity (CN-LOH) calls were identified. **B**–**D** The distribution of CNVs (**B**), SVs (**C**) and CN-LOH (**D**) represented on the *Y*-axes plotted across different chromosomes (chromosome number on the *X-*axis).

Using a single platform for unbiased SV detection enabled systematic detection of all types of somatic SVs at the same resolution without the need for germline DNA. A median coverage of >300x was achieved on all samples, permitting a detection sensitivity of ~10%. There were 4030 unique somatic variant calls, of which 383 (9.5%; included 222 CNVs; 161 SVs) were classified as “clinically significant” based on the overlap with a gene or genomic region of interest (Fig. 1). These included a broad spectrum of SV types involving both recurrent and novel regions/ genes of interest: CN aberrations (gains/losses), inversions, balanced and unbalanced rearrangements (intrachromosomal and interchromosomal, including three-way translocations). Specific patterns of chromosomal alterations such as dicentric and ring chromosomes, and isochromosomes were readily apparent. Visualization of multiple aberrations per sample enabled reconstruction of complex derivative chromosomes, and complex alterations of chromoanagenesis (chromothripsis, chromoplexy and chromoanasynthesis). In addition to gene-level alterations, structural alterations at the exon-level such as partial tandem duplications involving *KMT2A* (*KMT2A-PTD*) were detected. Copy-neutral loss-of-heterozygosity (CN-LOH), evaluated in a subset (*n* = 70) of patients frequently involved chromosome 11 followed by chromosomes 12 and 13. In total, OGM identified at least 1 clinically significant SV in 70 of 101 MDS patients (70%, compared to 62% by CBA), with a median of 1 (range, 0–47) per patient. Supplementary Fig. S2 shows representative examples SVs of different types.

### Comparison of SVs between OGM and chromosome banding analysis (CBA)

**Fig. 2:**
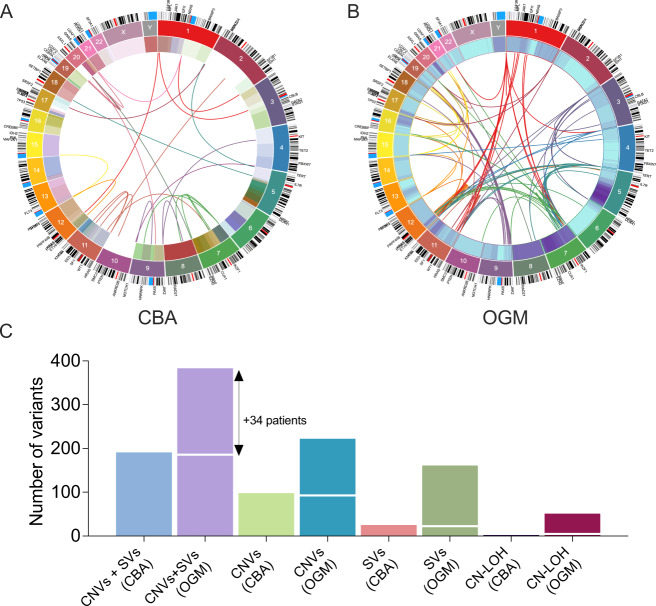
Comparison between the results of conventional chromosomal banding analysis (CBA) and optical genome mapping (OGM). **A**, **B** OGM detected nearly twice the number of clinically significant structural variants (SVs); circos plots illustrating SVs by CBA (**A**) versus OGM (**B**). **C** 189 of 383 clinically significant aberrations, included SVs and copy number variants (CNVs), were cryptic; in addition, a subset of patients had copy-neutral loss-of-heterozygosity (CN-LOH).

The distribution of chromosomal aberrations (CNVs, SVs and CN-LOH) detected by OGM compared to standard-of-care CBA is shown in Fig. 2. Even after excluding CN-LOH that cannot be detected by CBA, less than half (*n* = 189, 49%) of clinically significant SVs identified by OGM were detected by CBA. The remaining 224 (51%) clinically significant SVs, detected across 34 (34%) patients were cytogenetically silent, and only became apparent by OGM, either due to the sub-microscopic (below the limit of resolution) or cryptic nature of the alterations, or poor chromosomal morphology associated with CK. These included alterations such as segmental deletions in chromosomes 5q and 7q, *TP53* deletions and rearrangements, and *KMT2A* deletions, amplifications, and PTDs. By OGM, the proportion of MDS patients with normal karyotype (NK) decreased by 16% compared to CBA (not considering CN-LOH). Within the setting of NK, OGM uncovered cryptic rearrangements involving *MECOM*, *NUP98::PRRX2* (1 patient), *KMT2A-PTD* (in 2 patients), and deletions of genomic regions encompassing *TET2*, *KMT2A,* and *ETV6* (in 2 patients). Among those with CK, 17 (59%) patients not only demonstrated additional number of alterations, but a higher degree of genomic complexity than was apparent by CBA, and six (6%) patients showed chromoanagenesis (Supplementary Fig. S3). The higher resolution permitted clarification of uncertainties such as markers and additional material of unknown origin. In both patients with incomplete cytogenetic results, genome mapping generated successful sequence patterns (both showing NK) with adequate coverage and high-quality metric scores.

Conversely, in 96 of 99 patients, all clonal cytogenetic abnormalities detected by CBA and could be confirmed by FISH/CMA were also evident by OGM. The missed aberrations included the following, all noted in 2-3 metaphases (a) +8 seen in 3 of 20 metaphases [possibly due to proliferation bias during cell growth, since FISH demonstrated +8 in ~1.6% of cells, well below the detection threshold of OGM), (b) t(9;10)(q32;q24) and, (c) del(9)(q13q22).

### Integration of OGM and targeted NGS studies

**Fig. 3:**
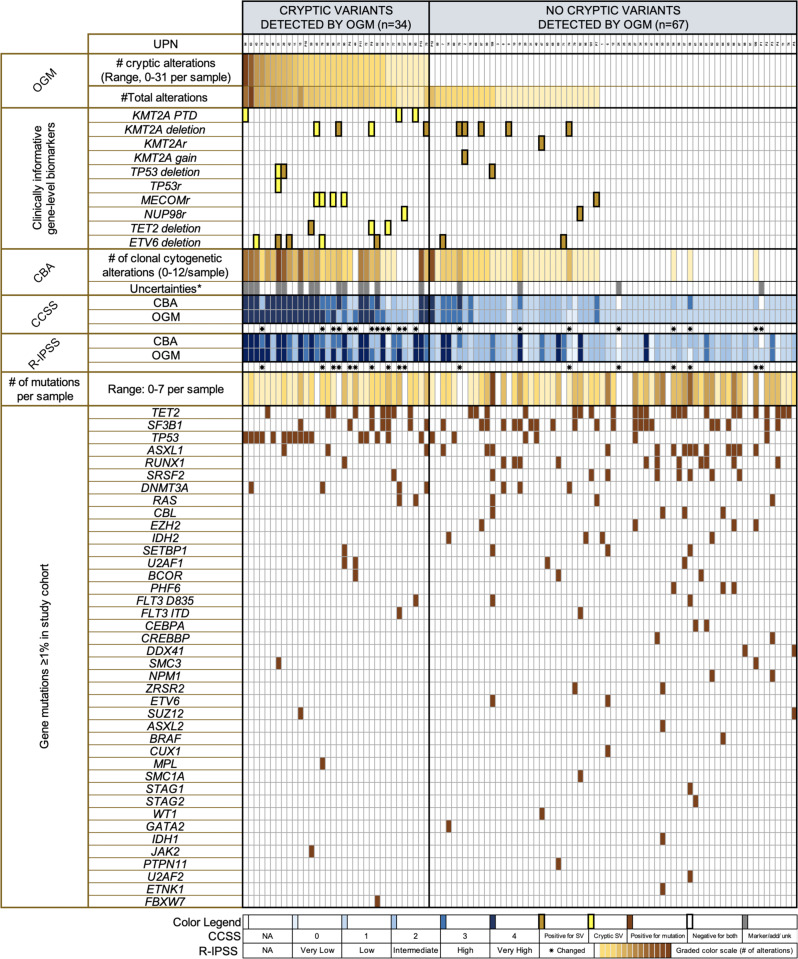
Relationship between cryptic variants detected by OGM alone and not by conventional chromosomal banding analysis (CBA) with findings from CBA and consequent changes of CCSS and R-IPSS risk categories and NGS based somatic mutation analysis in 101 MDS patients at baseline. The heatmap shows two clusters of patients: left panel represents patients with clinically significant cryptic variants detected by OGM, and right panel represents patients without clinically significant findings by OGM, compared to CBA. Patients are sorted left to right starting from the highest number of cryptic variants detected by OGM alone, followed by total OGM alterations, findings in key genes informative for MDS, CBA findings, including those with uncertainties in the karyotype such as markers and additional material of unknown significance. The resulting changes to the CCSS and R-IPSS risk categories are depicted by an asterisk. The rest of the heatmap shows the corresponding gene mutation data from a concomitant 81-gene NGS panel. Legend is provided at the bottom.

By targeted NGS alone, 13 (13%) patients had no somatic mutation. Among these, OGM identified three patients with *NUP98::PRRX2* and *MECOM* rearrangements. Among the rest, additional information of value included detection of *KMT2A* deletions in three additional patients and *ETV6* deletion in one patient. Integrating data from both NGS and OGM detected at least 1 driver alteration in 97 of 101 patients. Specifically, among 5 patients with no cytogenetic aberrations or mutations by CBA and targeted NGS, OGM detected a genetic abnormality in 1 patient. Combined mutation and SV profiling correctly identified biallelic *TP53* alterations in 3 patients (including a patient with wild-type *TP53* by NGS, further details below), and notable associations, such as double *RAS/FLT3* mutations in 2 of 3 *KMT2A*-PTD patients. The integrated findings of OGM, CBA and somatic mutations from each patient are shown in Fig. 3. Clinically significant SVs that were cryptic by CBA and detected by OGM alone were seen across all clinical, cytogenetic and mutational subsets, supporting the need for routine SVP in all MDS patients. The highest yield was seen in patients with higher number of cytogenetic abnormalities [CCSS score 3 (50%); 4 (90%)], R-IPSS [high (59%), very-high (70%)], and CK (~76%) [kappa: 0.776; SE = 0.049; *p* < 0.001]. Cryptic SVs were only identified in patients with ≤3 mutations detected by NGS, with the highest yield in patients with fewer mutations [1–2 per sample, ~48%; MDS with *TP53* (16, 73%) and *SF3B1* mutations (7, 25%)] (Supplementary Fig. S4).

### SVs involving clinically informative biomarkers of MDS

**Fig. 4:**
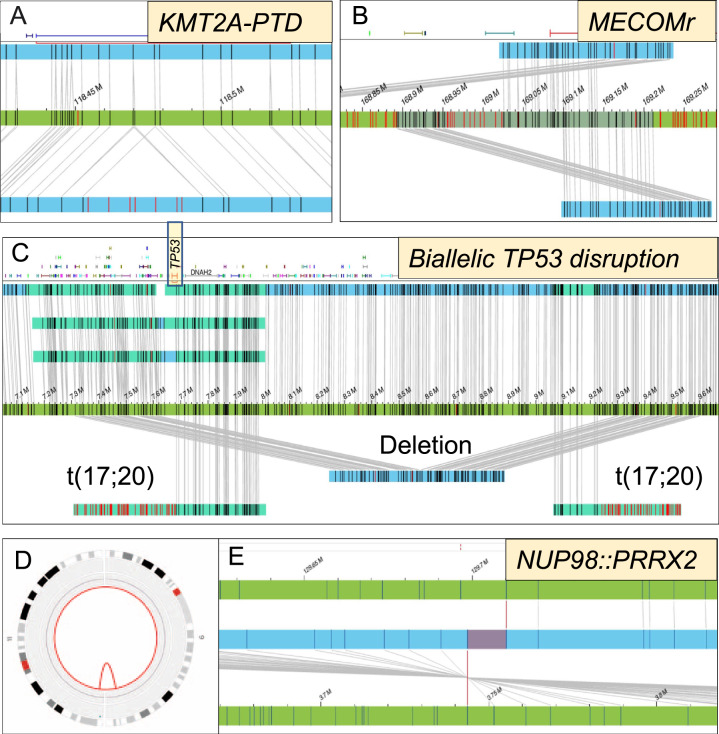
Precise mapping of SVs at gene/exon level allowed determination of the status of key clinically informative biomarkers in MDS. These included MDS patients with normal karyotype showing (**A**) partial tandem duplications affecting *KMT2A*, and (**B**) *MECOM* rearrangements of *MECOM*. MDS patient with wild-type *TP53* by NGS showing biallelic *TP53* alterations, deletion and translocation, both disrupting the gene (**C**) and MDS patient with normal karyotype showing cryptic *NUP98::PRRX*2 as shown in the circos plot comprising chromosomes 9 and 11 (**D**) and within the genome browser (**E**).

Precise mapping of SVs at gene-level, and in certain genes at the exon-level, allowed assessment of clinically informative biomarkers, without the need for confirmatory assays. In the context of MDS, we identified these as *TP53* for allele state, *MECOMr, NUP98r*, and *KMT2A* alterations. Of 22% *TP53* mutated patients, 16% had a single *TP53* mutation necessitating FISH and/or CMA testing to identify concomitant deletion and CN-LOH, respectively. OGM was able to correctly map concurrent *TP53* gene deletions in 2 of these patients, confirming biallelic *TP53* alterations. None had CN-LOH. In addition, one patient with wild-type *TP53* showed concomitant *TP53* deletion and translocation, both disrupting the gene (biallelic *TP53* alteration). There were patients with *NUP98* fusions (*n* = 2; 1 cryptic by CBA) and *MECOM* alterations (*n* = 5; 4/5 cryptic). Other alterations that are less well-defined with respect to clinical prognosis included deletions of *TET2* (*n* = 3; 2 cryptic) and *ETV6* (7; 2 cryptic). Within *KMT2A*, a multitude of structural alterations affecting the genomic regions of interest were detectable using a single platform. These included fusions (*n* = 1), deletions (*n* = 9; 2 cryptic), gains (*n* = 1) and partial tandem duplications (*n* = 3, all cryptic) involving exons 3 to 6, thereby expanding the scope of genetic analysis (Table 2; Fig. 4). The findings were confirmed using orthogonal assays.

**Table 2:** SVs involving key genes selected as clinically informative*/actionable** biomarkers in MDS detected using optical genome mapping.

Gene	Alteration(s)	Total^#^	Cryptic^##^ (Detected by OGM only)
KMT2A*	Deletion	9	2 (22%)
	Gain	1	0
	Rearrangement	1	0
	Partial tandem duplication	3	3 (100%)
MECOM*	Rearrangement	5	4 (80%)
NUP98*	Rearrangement	2	1 (50%)
TP53*	Deletion	3	1 (33%)
	Rearrangement	1	1 (100%)
TET2**	Deletion	3	2 (67%)
ETV6**	Deletion	7	2 (29%)

*Detection essential for prognosis or therapeutic decision making (when suspected or even when apparent by karyotype, this generally requires an additional assay for confirmation).

**Other aberrations, clinical significance not established.

^#^Detected by OGM or CBA and confirmed by an additional orthogonal assay.

^##^Detected by OGM only (not by CBA) and confirmed by an additional orthogonal assay.

OGM enabled detailed molecular characterization of rare SVs, such as identification of *ELL* as the gene partner for *KMT2A* in a case of *WT1* mutated MDS with t(11;19)(q23;p13.1). Direct visualization of molecules of t(9;11)(p23;q22) in an *SF3B1* mutated MDS revealed a rearrangement involving *SYTL2* gene along with a deletion of *KMT2A* (confirmed by FISH and chromosomal mapback). In another *SF3B1/RUNX1* mutated MDS, t(1;3)(p36;q21) could be mapped to *PRDM16::RPN1*.

### Changes in IPSS-R and clinical impact based on OGM results

**Fig. 5:**
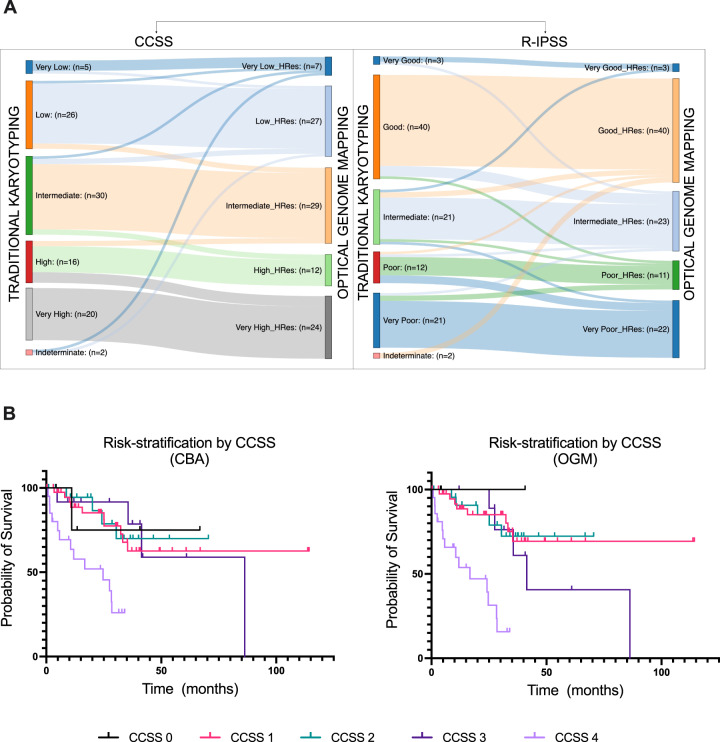
Clinical impact of optical genome mapping (OGM) results compared to conventional chromosome banding analysis (CBA). **A** Flow plots showing modifications to the CCSS (comprehensive cytogenetic scoring system) in 21% of patients and R-IPSS (revised international prognostic scoring system) in 17% of patients based on OGM results of the MDS study cohort. **B** Kaplan–Meier curves showing the overall survival of MDS patients stratified by CCSS cytogenetic-risk categories evaluated from CBA versus OGM results (OGM had a slightly improved prediction rate based on a slightly higher c-index).

To analyze the potential clinical impact of this information, we calculated and compared CCSS groups and R-IPSS categories by both techniques retrospectively. In the case of OGM, we recalculated the CCSS categories by alignment of multiple individual SVs to generate a predicted karyotype resulting in 224 unique SVs, with 94 (40%) being cryptic, detected across 34 patients. This upgraded the CCSS cytogenetic-risk in 12 patients (resulting in upgrade of R-IPSS in 9 patients) and downgraded in 7 patients (with downgrading of R-IPSS in 6 patients). Together with the generation of results for 2 patients with non-informative karyotype, OGM derived results led to modification of the cytogenetic-risk for 21% patients, leading to an overall change in R-IPSS risk category in 17 (17%) patients (Supplementary Table S4; Fig. 5).

CCSS change was primarily due to the change in the number of cytogenetic abnormalities, although the type of aberration and their combination also affected some cases. OGM upgraded CCSS scores in 12 MDS patients by uncovering one or more cryptic SV(s) that increased the overall number of cytogenetic abnormalities. This affected cases with NK, non-complex or complex karyotype with 3 abnormalities and resulted in CCSS upgrade by either 1 (in 9 patients) or 2 points (in 3 patients). More interestingly, OGM data downgraded the CCSS scores in 7 patients. In 5 patients, this was due to the higher resolution of OGM leading to clarification of karyotype in the setting of markers, additional material, and derivative chromosomes, which ultimately resulted in decrease in the overall number of cytogenetic abnormalities. In 2 patients, this was due to failure of OGM to detect low-level aberrations, and in 1 patient, a cryptic del(11q) in a NK MDS patient downgraded the CCSS from “good” to “very good”.

To capture the clinical impact of SVs that do not always change the CCSS/R-IPSS, we used an alternate approach. We defined an “actionable SV” as (1) prognostic, not recognized in the CCSS criteria (*KMT2A*-PTD, *TP53* alterations, knowledge essential to determine allele status) or (2) potential eligibility for an ongoing clinical trial (at the time of manuscript submission) such as *KMT2Ar*, *MECOMr*, or *NUP98r*. Well-established SVs defined in the CCSS were ignored, since these were already evaluated in the first approach. Based on this definition, 13 (13%) patients had an “actionable alteration” (9 with a positive finding) (Supplementary Table S5). When both CCSS change and actionable alterations were combined, OGM results could have informed data for prognosis/therapy in 28 (28%) patients.

Treatment information was available in 99 patients, of which 36 (36%) received disease modifying therapy for MDS [included hypomethylating agent (HMA) only (36%), HMA with investigational agents (50%), HMA with chemotherapy (6%) and chemotherapy only in 3 (8%) patients]. The remaining (64%) patients did not receive therapy (Table 1). Over a median follow-up of 34 months, the median overall survival was 86 months. Based on a higher concordance index, OGM had a slightly improved prediction of prognosis for both CCSS [0.7 (SE = 0.052) vs. 0.665 (SE = 0.053)] and R-IPSS [0.705 (SE = 0.046) vs. 0.69 (SE = 0.05)] compared to CBA, but the differences were not statistically significant. By univariate analysis, the following parameters associated with outcome: *SF3B1* mutation [*p* = 0.030; HR 0.343; CI: 0.130–0.901], *TP53* mutation [*p* = 0.001; HR 3.232; CI: 1.582–6.601], CCSS score by CBA [*p* = 0.001; HR 1.690; CI: 1.250–2.286] and CCSS by OGM [*p* < 0.001; HR 2.051; CI: 1.488–2.827]. By multivariable analysis, CCSS by OGM [*p* < 0.001; HR 1.956; CI: 1.367–2.798], but not CCSS by CBA), BM blasts [*p* = 0.026; HR 1.079; CI: 1.009–1.153] and *TP53* mutation [*p* = 0.040; HR 2.335; CI: 1.038–5.250] associated with prognosis (Table 3).

**Table 3:** Univariate and multivariable analysis for overall survival.

	Univariate Analysis			Backward MVA		
	*p*-value	HR	95% CI	*p*-value	HR	95% CI
Age	0.210	1.021	0.988–1.054			
Gender (female vs. male)	0.688	1.173	0.538–2.560			
Hgb	0.269	0.899	0.745–1.086			
MCV	0.143	0.976	0.945–1.008			
Platelet count	0.151	0.997	0.994–1.001			
ANC	0.621	0.959	0.810–1.134			
Therapy-related	0.275	1.538	0.710–3.331			
BM blasts	0.153	1.050	0.982–1.122	0.026	1.079	1.009–1.153
*SF3B1* mutation	0.030	0.343	0.130–0.901			
*TP53* mutation	0.001	3.232	1.582–6.601	0.040	2.335	1.038–5.250
CCSS score by CBA	0.001	1.690	1.250–2.286			
CCSS score by OGM	<0.001	2.051	1.488–2.827	<0.001	1.956	1.367–2.798
Disease modifying therapy vs. observation	0.586	0.819	0.400–1.679			

*ANC* Absolute Neutrophil count, *CBA* conventional chromosome banding analysis, *CCSS* Comprehensive cytogenetic scoring system, *CI* Confidence Interval, *HR* Hazard Ratio, *MCV* mean corpuscular volume, *MVA* multivariate analysis, *OGM* optical genome mapping.

## Discussion

In this study, we used OGM and targeted NGS to explore a spectrum of high-resolution (≥5000 bp) clinically significant SVs in MDS that escaped detection by standard-of-care CBA and targeted NGS designed for mutation analysis. The findings showed the lack of the current standard-of-care workflow to comprehensively evaluate the genomic landscape of MDS and provided insight into a plethora of SV data that could be potentially investigated for developing targeted therapeutics. Importantly, OGM data was complementary to NGS data.

Recent whole genome and whole transcriptomic sequencing studies have demonstrated the feasibility in detecting targeted SVs, defined in European Leukemia Network (ELN) risk-stratification for AML [43] and fusions associated with ALL [44]. However, unbiased whole genome SV detection remains a challenge, particularly within the setting of complex MDS genomes, due to the inherent technical limitations of sequencing. Short-read NGS, routinely used for mutation analysis, has a low sensitivity for detecting SVs due to challenges in the alignment of repetitive sequences that are hot-spots for SVs [18–20]. Current long-read (10–20 kbp) sequencing technologies have improved alignment but are prone for high systematic error rates [18, 21]. These problems could potentially be overcome using non-sequencing based OGM that allows for detection of all types of SVs by evaluating patterns generated by fluorophore tags labeled to sequence-specific motifs in ultra-long DNA molecules. Together with targeted deep NGS, this approach could provide high-resolution genomic data for precision therapeutics.

For this exploratory study, we selected MDS as a prototype due to the marked cytogenomic heterogeneity, immense clinical need for accurate genomic prognostication and expansion of therapeutic targets. Despite a stringent definition, we identified several cryptic SVs of prognostic and therapeutic potential that were not readily apparent by standard evaluation in 34% of patients. This information changed the CCSS score in 21% and R-IPSS risk in 17% (1 in ~7) patients. CCSS scoring by OGM independently associated with survival by multivariable analysis. Since CCSS and IPSS-R were developed in untreated patients at diagnosis and during the disease course, we explored the effect of therapy. By univariate analysis, therapy only slightly improved the survival (*p*-value 0.586, HR: 0.819), and did not reach statistical significance. Including therapy in multivariable analysis did not change the results. One likely interpretation for these data is that the number of patients is limited, and therefore these results are not a reflection on the efficacy of therapy.

In addition, OGM uncovered “actionable alterations” in 13 (13%) of patients not apparent by CBA, that did not necessarily change CCSS/R-IPSS but qualified patients for specific therapeutic strategies of potential benefit [UPN90: cryptic *MECOM* rearrangement; UPN51: biallelic *TP53* inactivation from a concurrent rearrangement and deletion, in the absence of a *TP53* mutation; both patients had no change to their CCSS] [45, 46].

Beyond CNVs that are frequent in MDS, the study revealed cryptic SVs potentially amenable to targeted therapeutics. For example, leukemias with *MECOMr, NUP98r, KMT2Ar*, and *KMT2A-PTD* are being explored as therapeutic targets within clinical trials [NCT04067336; NCT04811560] [10–15]. *KMT2A*-PTD is independently associated with poor prognosis and included in the IPSS-M model under development by IWG-PM. Due to the need for long-range PCR assays and difficultly in detection by NGS, limited laboratories perform *KMT2A-PTD* assessment. The frequency of *KMT2A-PTD* in our cohort (~3%) is similar to other reports [16, 47–49]. The finding of exon-level details suggests that OGM may bridge the gap between the large chromosomal alterations observed by CBA and small insertions/ deletions detectable by routine short-read NGS, which remain largely uninterrogated in routine work-up. This also emphasizes the complementary nature of OGM and NGS data, and co-operative interplay between SVs and mutations involving leukemogenic genes, as has been shown for *TP53*.

Finally, CN-LOH information can be extracted from OGM data within de novo assembly pipeline. Genomic regions of CN-LOH show consistent decrease in heterozygous SV calls compared to the level observed genome-wide in controls. CN-LOH can be easily distinguished from LOH resulting from a gain or a loss by reviewing the copy number changes using whole genome and circos plots. Although not included in the IPSS-R for calculation of the number of cytogenetic abnormalities, CN-LOH, specifically involving *TP53* gene is necessary for allele state determination and MDS prognostication using the latest IPSS-M [49].

The results of the study demonstrate that we are grossly under evaluating the degree of genomic aberrations using standard-of-care techniques. On top of significant increment in the diagnostic yield, OGM detected different types of SVs using one platform, rather than a multitude of techniques with different detection sensitivities and shortcomings. For example, CMA provides data on high-resolution CNA(s) and CN-LOH but lacks the ability to identify balanced translocations and inversions and resolve complex SVs [22]. With the emerging prognostic models that incorporate granular cytogenomic data (*TP53* allele state, *KMT2A-PTD)*, a single platform that enables comprehensive detection of all types of chromosomal alterations becomes invaluable. The ability to consolidate multiple clinical assays could also simplify the laboratory workflow, and lead to better utilization of technical expertise and resources. The average duration between sample receipt and interpretation of results was ~5 days (3–4-day run-time; 1 day for analysis). OGM can be readily implemented for comprehensive cytogenetic analysis without sophisticated bioinformatic support but would need a separate targeted NGS for single nucleotide level mutation analysis. Recent data showed WGS (60X coverage) as a feasible alternative to CBA, with simultaneous mutation data, both of which improved the risk-stratification of a subset of intermediate-risk AML [43]. When compared to targeted NGS (median ~1500X), mutation yield was compromised due to decreased depth of coverage, specifically in variants ≤35% VAF [50]. Considering those actionable mutations such as *IDH1/2* and *FLT3*, are often sub-clonal, and the current eligibility criteria for clinical trials is based on the presence or absence of mutations rather than the VAFs, this may pose specific challenges in enrollment for these patients [50–52]. Further, unbiased interpretation of all mutational variants by WGS, beyond those pertinent to ELN risk, will need simultaneous profiling of matched germline tissue [53].

The current study still underestimates the diagnostic yield of OGM and the clinical significance. For the sake of high stringency, we did not evaluate SVs between 500–5000 bp although detectable by de novo pipeline, SVs involving non-coding regions and those affecting genomic regions not implicated in myeloid cancers. R-IPSS model has not evaluated the clinical importance of some adverse prognostic SVs other than *MECOMr*, such as *NUP98r, KMT2Ar*, *KMT2A*-PTDs, chromoanagenesis due to either rarity or limited scope of traditional techniques. These need to be revisited in the future, within the context of mutations, using emerging technologies.

This is the first study to evaluate the clinical importance of SVP in the largest single center MDS cohort. However, there are some limitations. The study design is retrospective with an initial goal to explore the feasibility and clinical value of SVP. The findings need independent validation, on a large-scale in prospective multi-center cohorts within clinical trials. Unlike CBA, OGM cannot provide data at single-cell level (similar to all bulk nucleic-acid assays), which limits understanding of sub-clonal architecture. Nonetheless, this study presents evidence and opportunities for furthering efforts to facilitate a more precise genomic classification beyond current knowledge. Accumulating evidence from multiple such studies will guide their graded implementation into clinical laboratories and eventually set a new norm for routine high-resolution SVP [24].

Along similar lines, direct assessment of true biallelic inactivation of *TP53* requires analysis of mutation(s), CNV(s), SV(s) and CN-LOH at a single-cell resolution, which is not feasible using any of the routine bulk sequencing methodologies (OGM, targeting NGS, WES, WGS). This information needs to be inferred from quantitative variant fractions [16], and the “deleterious” nature of the rarer mutation(s) substantiated by computational algorithms or functional evidence [34]. Nevertheless, the superior ability of OGM in SV characterization offers advantages when combined with either targeted, whole genome or exome sequencing, especially within tumor suppressor genes. Within the current study, we identified a case of MDS with *TP53* biallelic inactivation by two different SVs (deletion and rearrangement), in the absence of a mutation. In another study, OGM outperformed WGS/WES in SV detection within repetitive and difficult to map genomic regions such as 22q11.2 due to the use of ultra-long DNA molecules [54]. OGM also enabled detection of unique types of SVs that can be missed by WES/WGS. In a separate study, OGM identified an intronic insertion (retrotransposon element) within the *SMARCB1* tumor suppressor gene as the genetic cause of a congenital atypical rhabdoid tumor. This insertion was missed by CMA, WES, and WGS [55]. Therefore, it is important to be cognizant of the complementary nature of different genomic technologies [56].

In summary, our data provide support that pan-genomic profiling by combined OGM and targeted deep NGS, improves risk-stratification in MDS by uncovering a gamut of high-resolution actionable genomic aberrations.

## Supplementary information

Supplementary Material

## Data Availability

The datasets generated during and/or analyzed during this study are not publicly available due to patient privacy concerns but are available from the corresponding author upon request.
