# Supplementary material for: High-resolution structural variant profiling of myelodysplastic syndromes by optical genome mapping uncovers cryptic aberrations of prognostic and therapeutic significance
Source: Leukemia. 2022 Aug 1;36(9):2306–16. doi: 10.1038/s41375-022-01652-8 (PMC9417987; doi:10.1038/s41375-022-01652-8)

SUPPLEMENTARY MATERIAL

High-Resolution Structural Variant Profiling of Myelodysplastic Syndromes by Optical Genome Mapping Uncovers Cryptic Aberrations of Prognostic and Therapeutic Significance

Hui Yang[1], Guillermo Garcia-Manero[1], Koji Sasaki[1], Guillermo Montalban-Bravo[1], Zhenya Tang[2], Yue Wei[1], Tapan Kadia[1], Kelly Chien[1], Diana Rush[3], Ha Nguyen[3], Awdesh Kalia[3], Manjunath Nimmakayalu[3], Carlos Bueso-Ramos[2], Hagop Kantarjian[1], L. Jeffrey Medeiros[2], Rajyalakshmi Luthra[2], Rashmi Kanagal-Shamanna[2†]

Departments of [1]Leukemia, [2]Hematopathology, [3]School of Health Professions, The University of Texas M.D. Anderson Cancer Center, Houston, Texas

[†]**Correspondence:**

Rashmi Kanagal-Shamanna, MD

Department of Hematopathology & Molecular Diagnostics

The University of Texas MD Anderson Cancer Center

6565 MD Anderson Boulevard (Z3. 5044) I Houston I TX, USA 77030

Phone: (713) 745-4947

E-mail: RKanagal@mdanderson.org

Short Title: Genome-Wide Structural Variant Profiling in MDS

MATERIALS AND METHODS

Targeted NGS for somatic mutations

All patients underwent amplicon-based targeted mutation analysis by next-generation sequencing using an 81-gene panel **(Supplementary Table S1)** within the CLIA-certified Molecular Diagnostics Laboratory as previously described [1-3]. Briefly, 250-500 ng of genomic DNA extracted from whole mononuclear cells from fresh BM aspirates was used for library preparation using HaloPlex chemistry (Agilent Technologies, Santa Clara, CA). Multiplexed bi-directional sequencing (total of ~310 cycles) was performed on a MiSeq V3 300 cycle kit (Illumina, SanDiego, CA) using appropriate controls. Sequencing data was processed through Miseq reporter (Illumina) and Surecall 3.0 software (Agilent technologies) . A minimum of 2% VAF with adequate bidirectional coverage (250X minimum; median: 1500X) was required for variant calling. Since matched germline samples were not sequenced, the somatic nature of the variants was inferred based on the VAFs, evidence from the literature and online databases such as COSMIC and data from our institutional cohort. Variants reported in the Exome Aggregation Consortium [ExAC], dbSNP 137/138, and 1000 Genomes Project databases were excluded. *FLT3* ITD mutations were evaluated by PCR-based capillary electrophoresis.

Cytogenetic analysis

Conventional karyotypic studies were performed from metaphase spread prepared from unstimulated 24-hour and 48-hour BM aspirate cultures using standard G-banding techniques. At least 20 metaphases were evaluated and reported according to the 2016 International System for Human Cytogenetic Nomenclature [4, 5]. FISH analysis for deletions of chromosomes 5/5q, 7/7q, +8, -17p/*TP53*, del(20q), *KMT2A*, *MECOM* etc was performed on freshly harvested aspirate smears or cultured cells using standard techniques [6]. A total of 200 interphase nuclei were analyzed.

Chromosomal Microarray (CMA)

CMA was done on Agilent's custom-designed whole-genome SurePrint G3 dual-color array (4x180K chip, CCMC), with 60-mer probes [a total of 120,000 comparative genomic hybridization probes plus 60,000 single-nucleotide polymorphism probes, with ~13 Kb genome-wide median probe spacing; the probes span >500 cancer genes and 4130 cancer-associated genomic regions]. Briefly, 500 ng of genomic DNA extracted from BM aspirate samples underwent restriction enzyme digestion using Alu and Rsal, followed by Cy5-dUTP labeling using the Agilent

Genomic DNA Labeling Kit Plus. For control, reference human (female) DNA (Promega Corporation, Madison, WI) was labeled with Cy3-dUTP. This was followed by hybridization per manufacturer's recommendations. The slides were scanned using a high-resolution microarray scanner (Agilent Technologies, CA). The data analysis was done using CytoGenomics software. On a subset of cases, SNP array was performed on the Infinium CytoSNP-850K Beadchip Arrays (Illumina, Inc.) using 200 ng of genomic DNA. The data was generated using iscan and converted to gtc file using Beeline V2.0, and subsequently evaluated using NxClinical v6.1 (BioDiscovery Inc.).

Optical Genome Mapping (OGM)

DNA extraction, labeling and chip loading

We used BM mononuclear cells (BMMNCs) isolated from patient samples by ficoll density-gradient centrifugation that were either stored as viable cells in liquid nitrogen or cell pellets at -80°C within 3 days of collection. Ultra-high molecular weight (UHMW) genomic DNA (gDNA) was extracted from 1.5 million cells following manufacturer's protocols (Bionano Prep SP Blood and Cell DNA Isolation Kit; Bionano Genomics, San Diego, CA). Briefly, after thawing in 37°C water bath, cryopreserved BMMNCs were washed with DNA stabilizing buffer (Bionano Genomics, San Diego, CA) to get the cell pellets. Cell pellet was re-suspended in 40ul DNA stabilizing buffer, lysed and digested with proteinase K, RNase A and buffer LBB (Bionano Genomics, San Diego, CA). After PMSF treatment (Sigma-Aldrich, St. Louis, MO), nanobind paramagnetic disk (Bionano Genomics, San Diego, CA) was added to the solution, and mixed with isopropanol to precipitate the gDNA. The precipitated gDNA, bound to the nanobind disk, was washed with WB1 and WB2 (Bionano Genomics, San Diego, CA). After transferring the disk to a new tube, buffer EB (Bionano Genomics, San Diego, CA) was added to elute the gDNA from the disk. The gDNA was equilibrated overnight at room temperature to homogenize and subsequently quantified using Qubit BR dsDNA assay kit with Qubit Fluorometer (ThermoFisher Scientific, CA).

Sequence-specific Direct label and stain (DLS) technique was used for labeling the UHMW gDNA following manufacturer's protocols (Bionano Prep DLS Labeling Kit; Bionano Genomics, San Diego, CA). Briefly, Direct Labeling Enzyme 1 (DLE-1) reaction was carried out using 750 ng of purified gDNA to tag a specific 6bp sequence (CTTAAG) with a DL-green fluorophore (~15 times per 100 kb). Following puregene proteinase K (Qiagen, Hilden, Germany) digestion and DL-Green clean-up using DLS membrane in DLS 24-well plate, the DL-green

labeled gDNA was mixed with DNA stain, stained overnight at room temperature for backbone visualization and quantified using Qubit HS dsDNA assay kit (ThermoFisher Scientific, CA). The fluorescent-labeled gDNA molecules were loaded on a Saphyr chip G2.3, and linear double stranded gDNA molecules passing across nanochannels were imaged sequentially on a Saphyr instrument (Bionano Genomics, San Diego, CA). Effective genome coverage of approximately 300X was achieved for every tested sample (1,300 GB data per sample), in theory, enabling detection of aberrations at a 5% allele frequency (equivalent to aberrations in 10% of cells when heterozygous). Standard run quality control parameters [total DNA (≥150 kbp), N50 (≥150kbp), map rate (≥150 kbp), effective coverage (>300x) and average label density (per 100kbp)] were evaluated per manufacturer's guidelines.

Data Analysis and Variant Filtering

Data was analyzed using Bionano Access (Bionano Genomics, San Diego, CA) using Genome Reference Consortium GRCh38/hg38 as the reference. Identification of SVs was based on discrepant alignment between the molecules of the sample (following assembly of consensus genome maps from molecule clusters showing the same SV) and reference (GRCh38/hg38), with no assumption about ploidy. For fractional CN analysis, following alignment of molecules/labels against GRCh38/hg38, sample's raw label coverage was normalized against relative coverage from normal human controls, segmented and baseline CN state was estimated [mode of coverage of all labels; coverage in sex chromosomes was halved if chromosome Y molecules were present]. CN states of segmented genomic intervals were assessed for significant increase/decrease from the baseline.

Data analysis was performed in a single-blinded fashion independently by 2 users using *de novo* (DN; for detection of SVs>500bp), rare variant (RV; for detection of SVs>5,000bp) and copy number pipelines (CN; for capturing large CNVs >500,000bp potentially missed by SV algorithms). RV pipeline enabled detection of SVs occurring at low allelic fractions (~10%). Based on prior sensitivity studies using simulations, serial dilutions and cell lines, a detection sensitivity of ~95% for SVs with an allele fraction of ~10% was achieved (data not shown). DN pipeline was primarily used for CN-LOH assessment and confirmation of SV calls>5,000 bp detected by RV pipeline; SV calls between 500 bp and 5,000 bp were not included for this study.

For variant filtering, as a first step, we used the recommended size and confidence score filters for each of the three pipelines for to generate a list of high confidence SVs and copy number

variants for analysis described elsewhere (**Supplementary Table S2**) [7-10]. For the second step, we used the OGM data generated from 200 healthy individuals to select only the rare variants that represent pathogenic somatic alterations by filtering out the variants seen in normal population. Finally, as a third step, in order to select clinically significant SVs, we selected variants that overlapped the coding region of a gene/ chromosome locus implicated in myeloid neoplasm, adapted from the publicly available myeloid neoplasm-specific gene list (created through a collaboration between the Cancer Genomics Consortium and the Mayo Clinic (Genomics of Oncology Annotation Team: https://www.cancergenomics.org/gene_lists.php) and in-house 81-gene NGS mutation panel (**Supplementary Table S3**). The final interpretation of every call was made after visualizing the sample molecules for changes in the sequence patterns compared to the reference.

Limit of Detection and Reproducibility/ Precision

SV detection by OGM is dependent on 3 inter-related parameters: size (bp), type and clonal burden of the aberrations that can influence the limit of detection (LOD). Evaluation of LOD is challenging due to the limited number of cells available from patient samples for serial dilution. Therefore, we used other evidence to support this. Since we set RV pipeline threshold of >5000 bp for SV calling for this study, we relied on white paper RV pipeline data to generate this information using dilutions of different types of calls at 300X effective coverage of simulated data. The analysis showed that deletions (≥5000 bp), duplications (>150 kbp), insertions (5-50 kbp), inversions (>70 kbp) and translocations were detectable at 5% variant allele frequency at least 90% of the time using the RV pipeline [10]. Additionally, independent assessment of the LOD for CNVs and SVs was recently investigated in patient samples. Sahajpal et al evaluated LOD for deletions, duplications, aneuploidy and translocations, and showed that all variants were detected in triplicate at 5% allele fraction (10% of cells) [11]. Further, limit of detection could vary in the presence of other cytogenetic abnormalities, especially a complex karyotype and hence, more systematic studies using multiple samples with 2 or more concomitant aberrations are needed.

To confirm the reproducibility/ precision, over the study interval, we performed duplicate testing on aliquots from 4 different patient samples, 1 with a normal karyotype and the remaining 3 samples showing a variety of aberrations including deletion, duplication and translocations [del(5q), del(7q), dup(1p36), inv(3)(q21q26.2), -Y among others] on different runs, and analyzed them at separate time points using the same settings for RVP and DVP. The results were 100% concordant.

Statistical Analysis

Overall survival (OS) was calculated from the time from diagnosis to death or the last follow-up date. Patients who were alive at their last follow-up were censored on that date. The Kaplan-Meier product limit method was used to estimate the median OS for each parameter. Univariate Cox proportional hazards regression analysis was used to identify association of each of the variables with OS, followed by multivariate analysis. Prognostic fitness of cytogenetic risk calculated from CBA and OGM were compared using Harrell's concordance index.

REFERENCES

1. Kanagal-Shamanna R, Singh RR, Routbort MJ, Patel KP, Medeiros LJ, Luthra R. Principles of analytical validation of next-generation sequencing based mutational analysis for hematologic neoplasms in a CLIA-certified laboratory. Expert Rev Mol Diagn 2016;16:461-472.

2. Kanagal-Shamanna R, Montalban-Bravo G, Katsonis P, Sasaki K, Class CA, Jabbour E, *et al.* Evolutionary action score identifies a subset of TP53 mutated myelodysplastic syndrome with favorable prognosis. Blood Cancer J 2021;11:52.

3. Kanagal-Shamanna R, Montalban-Bravo G, Sasaki K, Darbaniyan F, Jabbour E, Bueso-Ramos C, *et al.* Only SF3B1 mutation involving K700E independently predicts overall survival in myelodysplastic syndromes. Cancer 2021;127:3552-3565.

4. Kanagal-Shamanna R, Bueso-Ramos CE, Barkoh B, Lu G, Wang S, Garcia-Manero G, *et al.* Myeloid neoplasms with isolated isochromosome 17q represent a clinicopathologic entity associated with myelodysplastic/myeloproliferative features, a high risk of leukemic transformation, and wild-type TP53. Cancer 2012;118:2879-2888.

5. McGowan-Jordan J. *ISCN 2016: An International System for Human Cytogenomic Nomenclature (2016); Recommendations of the International Standing Human Committee on Human Cytogenomic Nomenclature Including New Sequence-based Cytogenomic*. Karger, 2016.

6. Kanagal-Shamanna R, Yin CC, Miranda RN, Bueso-Ramos CE, Wang XI, Muddasani R, *et al.* Therapy-related myeloid neoplasms with isolated del(20q): comparison with cases of de novo myelodysplastic syndrome with del(20q). Cancer Genet 2013;206:42-46.

7. Mantere T, Neveling K, Pebrel-Richard C, Benoist M, van der Zande G, Kater-Baats E, *et al.* Optical genome mapping enables constitutional chromosomal aberration detection. Am J Hum Genet 2021;108:1409-1422.

8. Neveling K, Mantere T, Vermeulen S, Oorsprong M, van Beek R, Kater-Baats E, *et al.* Next-generation cytogenetics: Comprehensive assessment of 52 hematological malignancy genomes by optical genome mapping. Am J Hum Genet 2021;108:1423-1435.

9. Rack K, De Bie J, Ameye G, Gielen O, Demeyer S, Cools J, *et al.* Optimizing the diagnostic workflow for acute lymphoblastic leukemia by optical genome mapping. Am J Hematol 2022;97:548-561.

10. Genomics B. Bionano Solve Theory of Operation: Structural Variant Calling. Bionano Genomics 2020.

11. Sahajpal NS, Mondal AK, Tvrdik T, Hauenstein J, Shi H, Deeb KK, *et al.* Optical Genome Mapping: Clinical Validation and Diagnostic Utility for Enhanced Cytogenomic Analysis of Hematological Neoplasms. medRxiv 2022;2022.2003.2014.22272363.

Supplementary Table S1. List of genomic regions interrogated by the 81-gene panel NGS

**Gene**	**Exons (codons) tested**
ANKRD26 (NM_014915)	1 (1-6)
ASXL1 (NM_015338)	11-12 (362-892), 12 (897-1290), 12 (1299-1436), 12 (1450-1542)
ASXL2 (NM_018263)	11 (381-579), 11-12 (582-1436)
BCOR (NM_017745)	2-4 (1-511), 4-6 (515-1080), 7 (1122-1168), 7 (1080-1088), 7 (1090-1099), 8-12 (1168-1547), 13-15 (1550-1644), 15 (1663-1722)
BCORL1 (NM_021946)	1-6 (1-1261), 6 (1292-1323), 6-11 (1326-1600), 11-12 (1606-1700), 12 (1706-1712)
BRAF (NM_004333)	11 (439-478), 15 (581-620)
BRINP3 (NM_199051)	2-6 (1-321), 7-8 (327-767)
CALR (NM_004343)	9 (352-418)
CBL (NM_005188)	7-9 (336-477)
CBLB (NM_170662)	7 (282-287), 7-9 (297-397), 10 (402-469)
CBLC (NM_012116)	7-9 (336-454), 10 (465-475)
CEBPA (NM_004364)	1 (1-96), 1 (249-358), 1 (215-244), 1 (128-175), 1 (178-201)
CREBBP (NM_004380)	1-8 (1-608), 9-22 (615-1286), 22-31 (1293-1943), 31 (1950-2443)
CSF3R (NM_156039)	14 (575-622), 17 (681-800), 17 (813-864)
CUX1 (NM_181552)	2-6 (11-172), 6-9 (174-241), 10-12 (248-359), 13-14 (368-408)
DDX41 (NM_016222)	1-11 (1-410), 12-17 (415-623)
DNMT3A (NM_022552)	8-22 (286-862), 23 (866-913)
EED (NM_003797)	1-2 (1-69), 2-8 (71-287), 9-12 (289-442)
ELANE (NM_001972)	1-2 (1-48), 2 (69-75), 3-5 (102-268)
ETNK1 (NM_018638)	3 (228-275)
ETV6 (NM_001987)	1-4 (1-147), 5-6 (155-378), 7-8 (385-453)
EZH2 (NM_004456)	2-4 (1-121), 5 (158), 6 (162-168), 6 (188-205), 7 (209-217), 8-13 (243-512), 14-15 (516-613), 15-19 (616-732), 20 (752)
FBXW7 (NM_033632)	9-12 (413-708)
FLT3 (NM_004119)	11-20 (437-847)
GATA1 (NM_002049)	2-3 (1-84)
GATA2 (NM_032638)	2-5 (1-377), 5-6 (379-481)
GFI1 (NM_005263)	2 (2-39)
GNAS (NM_000516)	8 (200-202), 11 (315-324)
HNRNPK (NM_002140)	3-7 (1-95), 7 (101-108), 8-11 (111-283), 11-17 (285-465)
HRAS (NM_005343)	2-3 (1-70), 3 (74-82), 3-4 (86-150)
IDH1 (NM_005896)	4 (132-133)
IDH2 (NM_002168)	4 (125-178)
IKZF1 (NM_006060)	2-8 (1-431), 8 (445), 8 (481-518)

IL2RG (NM_000206)	1-8 (1-370)
IL7R (NM_002185)	5-7 (180-292)
JAK1 (NM_002227)	3-22 (3-1023), 22-24 (1026-1123)
JAK2 (NM_004972)	10 (405-442), 12-14 (505-622), 16 (665-711), 18 (762-802)
JAK3 (NM_000215)	2-23 (1-1069)
KDM6A (NM_021140)	1-9 (1-223), 9-19 (228-971), 19-21 (977-1070), 22-29 (1080-1402)
KIT (NM_000222)	8-9 (411-514), 11 (550-592), 17 (788-828)
KMT2A (NM_005933)	2 (145-168), 3-4 (176-1075), 4 (1081-1112), 5 (1117-1184), 6 (1190-1212), 7 (1224-1325), 8-10 (1338-1440), 11-13 (1445-1560), 14-15 (1566-1665), 27 (2186-2195), 27 (2201-2355), 27 (2373-3215), 27 (3223-3324), 27 (3339), 27 (3342-3575)
KRAS (NM_004985)	2-4 (1-150)
MAP2K1 (NM_002755)	2 (27-90), 3 (98-146)
MPL (NM_005373)	10 (490-522), 12 (552-636)
NF1 (NM_001042492)	2-5 (21-189), 6 (201-218), 8-9 (244-354), 10-11 (359-399), 11-13 (418-467), 13-14 (478-547), 16-17 (574-667), 18 (674-728), 18-22 (746-992), 23-24 (1003-1066), 25-26 (1082-1146), 26-30 (1160-1370), 31-34 (1382-1494), 34 (1512-1518), 35 (1526-1549), 35 (1564-1575), 36-38 (1602-1868), 39 (1870-1884), 39-40 (1886-1946), 40-43 (1953-2210), 44-47 (2215-2322), 47-49 (2325-2437), 50-51 (2441-2492), 51-52 (2495-2555), 53-58 (2580-2840)
NOTCH1 (NM_017617)	26 (1529-1600), 26-28 (1604-1795), 34 (2061-2286), 34 (2290-2556), 34 (2061-2286), 34 (2290-2556)
NPM1 (NM_002520)	11 (283-295)
NRAS (NM_002524)	2-4 (1-150)
PAX5 (NM_016734)	1-10 (1-392)
PHF6 (NM_032458)	2-3 (1-78), 4-10 (81-366)
PIGA (NM_002641)	2 (1-6), 2-6 (16-485)
PML (NM_033238)	3 (201-255)
PRPF40B (NM_001031698)	2-19 (2-609), 19-20 (611-658), 20-26 (661-893)
PTEN (NM_000314)	7 (212-267), 8 (310-339)
PTPN11 (NM_002834)	3-4 (46-125), 7 (253-285), 12 (460-462), 12-13 (465-533)
RAD21 (NM_006265)	2-3 (1-82), 4-13 (92-568), 14 (612-632), 14 (569-597), 14 (599-601)
RARA (NM_000964)	6-7 (211-338)
RUNX1 (NM_001754)	2-9 (1-438), 9 (456-474)
SETBP1 (NM_015559)	4 (838-885)
SF1 (NM_004630)	1-13 (1-640)
SF3A1 (NM_005877)	1-9 (1-424), 9-12 (427-641), 13-16 (651-794)
SF3B1 (NM_012433)	13 (574-599), 14 (603-638), 14-16 (649-790)
SH2B3 (NM_005475)	2 (1-120), 2 (132-175), 2 (189-205), 2-8 (210-576)
SMC1A (NM_006306)	1-25 (1-1234)

SMC3 (NM_005445)	1 (1-5), 2-6 (19-110), 6-16 (113-504), 16-17 (507-580), 17-20 (591-728), 20 (736-756), 21-25 (762-975), 25-27 (984-1150), 28-29 (1159-1217)
SRSF2 (NM_003016)	1 (1-38), 1 (45-121)
STAG1 (NM_005862)	2 (1-5), 3-7 (10-188), 7-8 (194-276), 9-12 (279-392), 13-20 (402-703), 21-22 (724-738), 22-24 (740-809), 24-27 (825-953), 27-28 (955-1022), 29-34 (1029-1259)
STAG2 (NM_006603)	2-15 (1-512), 16-33 (541-1232)
STAT3 (NM_139276)	17 (489-503), 17-22 (506-715)
STAT5A (NM_003152)	3-6 (1-177), 6-7 (181-206), 8-9 (264-286), 9-20 (315-795)
STAT5B (NM_012448)	16 (636-693)
SUZ12 (NM_015355)	1 (20-44), 1 (46-92), 4-5 (129-169), 7-16 (198-740)
TERT (NM_198253)	1 (1-24), 2 (74-172), 2-4 (246-630), 4-5 (633-677), 6-8 (711-800), 8-16 (805-1133)
TET2 (NM_001127208)	3 (1-77), 3 (91-93), 3 (98-826), 3 (829-853), 3-10 (876-1455), 10-11 (1465-2003)
TP53 (NM_000546)	2 (1-25), 4-11 (80-394)
U2AF1 (NM_006758)	2 (15-44), 6 (117-161)
U2AF2 (NM_007279)	1 (1-17), 3-5 (62-161), 6-12 (163-473)
WT1 (NM_024426)	1 (122-216), 1 (2-59), 1 (72-91), 2-10 (216-518)
ZRSR2 (NM_005089)	1-3 (1-68), 4 (71-90), 6-8 (134-257), 9 (260-263), 9-11 (268-435), 11 (440-483)

Supplementary Table S2. Size and confidence score filters to generate a list of high confidence SVs and copy number variants for analysis.

BED SV Overlap Precision (Kbp)	12
BED CNV Overlap Precision (Kbp)	500
SV Masking Filter	All
Copy Number Type	All
Copy Number Confidence	0.99
Copy Number Min Size (bp)	500000
Self-Molecule Count	3
% in Control	1
% in Control for Enzyme	1
Found in Self Molecules	Yes
Overlap Genes	All
Insertion	0
Deletion	0
Inversion	0.7
Duplication	-1
Intra-Translocation	0.3
Inter-Translocation	0.65
CNV fractional analysis	
Deletions	<1.8
Duplications	>2.2

Supplementary Table S3. List of composite gene list for determination of clinical significance of SV calls from optical genome mapping. This was generated by combining the publicly available myeloid neoplasm-specific gene list created through a collaboration between the Cancer Genomics Consortium and the Mayo Clinic Genomics of Oncology Annotation Team and our in-house 81-gene NGS mutation panel.

ABL1	*ERCC2*	*LLGL2*	*RAD21*
ACSL6	*ETFDH*	*LUC7L2*	*RARA*
ADRB2	*ETNK1*	*MAL*	*RB1*
AEBP2	*ETV6*	*MAP2K1*	*RBBP6*
ANKRD26	*EZH2*	*MDS2*	*RBM15*
APC	*FAT1*	*MECOM*	*RBM22*
AR	*FAT4*	*MGA*	*RCOR1*
ARHGAP26	*FBXW7*	*MKL1*	*RELN*
ARNT	*FGFR1*	*MLF1*	*RPL22*
ASXL1	*FGFR1OP*	*MLLT1*	*RPN1*
ASXL2	*FHIT*	*MLLT10*	*RPS14*
BAALC	*FIP1L1*	*MLLT11*	*RUNX1*
BCOR	*FLT3*	*MLLT3*	*RUNX1T1*
BCORL1	*FOXN3*	*MLLT4*	*SALL3*
BCR	*FOXP1*	*MPL*	*SBDS*
BRAF	*GATA1*	*MSI2*	*SETBP1*
BRINP3	*GATA2*	*MYB*	*SF1*
CALR	*GFI1*	*MYBL2*	*SF3A1*
CBFB	*GNAS*	*MYC*	*SF3B1*
CBL	*GRIA1*	*MYH11*	*SGK2*
CBLB	*GUCY1B3*	*MYO18A*	*SH2B3*
CBLC	*HBS1L*	*NF1*	*SMC1A*
CCDC6	*HIP1*	*NIN*	*SMC3*
CDKN1B	*HMGA2*	*NOTCH1*	*SOCS2*
CDKN2A	*HNRNPK*	*NPM1*	*SOCS3*
CEBPA	*HOXA11*	*NR3C1*	*SPARC*
CHIC2	*HOXD11*	*NRAS*	*SPECC1*
CNTRL	*HOXD13*	*NSD1*	*SRSF2*
CREBBP	*HRAS*	*NUP214*	*SSBP2*
CRLF2	*HSPA9*	*NUP98*	*STAG1*
CSF1R	*IDH1*	*P2RY8*	*STAG2*
CSF3R	*IDH2*	*PAX5*	*STAT3*
CSMD2	*IKZF1*	*PCM1*	*STAT5A*
CSNK1A1	*IL2RG*	*PDE4DIP*	*STAT5B*

CTNNA1	*IL7R*	*PDGFRA*	*SUZ12*
CUX1	*INPP5D*	*PDGFRB*	*SYK*
DDX41	*JAK1*	*PER1*	*TERT*
DEK	*JAK2*	*PHF6*	*TES*
DHX15	*JAK3*	*PICALM*	*TET1*
DHX30	*JARID2*	*PIGA*	*TET2*
DIAPH1	*KAT6A*	*PML*	*TP53*
DKC1	*KDM6A*	*PPM1D*	*TPO*
DNMT1	*KIT*	*PRDM16*	*TRPS1*
DNMT3A	*KMT2A*	*PRKG1*	*U2AF1*
EED	*KMT2C*	*PRPF40B*	*U2AF2*
EGLN1	*KMT2E*	*PRPF8*	*UTY*
EGR1	*KRAS*	*PTEN*	*WT1*
ELANE	*L3MBTL1*	*PTPN11*	*ZC3H18*
ENAH	*LAMB1*	*PTPRT*	*ZMYM2*
EP300	*LINC00982*	*RABEP1*	*ZRSR2*

Supplementary Table S4. Tabular view comparing the CCSS and R-IPSS scores of MDS patients evaluated using conventional chromosome banding analysis (CBA) compared to optical genome mapping

		Optical Genome Mapping					
	**CCSS**	**Very Good**	**Good**	**Intermediate**	**Poor**	**Very Poor**	
Conventional karyotyping	**Very Good**	2	0	1	0	0	3
	**Good**	0	35	4	1	0	40
	**Intermediate**	1	2	16	1	1	21
	**Poor**	0	1	1	7	3	12
	**Very Poor**	0	0	1	2	18	21
	**Indeterminate**	0	2	0	0	0	2
		3	40	23	11	22	**Total**

		Optical Genome Mapping					
	**IPSS-R**	**Very Low**	**Low**	**Intermediate**	**High**	**Very High**	
Conventional karyotyping	**Very Low**	4	1	0	0	0	5
	**Low**	1	23	2	0	0	26
	**Intermediate**	1	2	25	2	0	30
	**High**	0	0	2	10	4	16
	**Very High**	0	0	0	0	20	20
	**Indeterminate**	1	1	0	0	0	2
		7	27	29	12	24	**Total**

Supplementary Table S5. List of MDS patients where OGM detected actionable alterations, defined as any alteration that changes the prognostic classification or therapy (including eligible for a clinical trial).

ID	Actionable SV*	CCSS change	Significance	Other**
1	*MECOM* rearrangement	No	∗ R-IPSS prognostication (*MECOM*)<br>∗ Clinical trial enrollment	
2	Absence of *TP53* alteration [add(17p) by karyotyping]	No	∗ Prognostication (monoallelic vs. multiallelic *TP53* alteration)	∗ Gene-level (*ETV6*) characterization of del(12p)<br>∗ Chromoanagenesis<br>∗ Resolution of “add” and “mar”<br>∗ Absent *KMT2A* deletion
3	Biallelic *TP53* alteration with wild-type *TP53* (deletion and translocation)	No	∗ Prognostication (monoallelic vs. multiallelic *TP53* alteration)	∗ *ETV6* deletion<br>∗ No *MECOM*<br>∗ Del(7)<br>∗ Dup(6)<br>∗ Chromoanagenesis
4	Cryptic *NUP98::NSD1* rearrangement	Yes	∗ Clinical trial enrollment	
5	*KMT2A* partial tandem duplication	Yes	∗ Prognostication	
6	*KMT2A* deletion	Yes	∗ Prognostication	∗ *TET2* deletion<br>∗ Absence of *KMT2A* fusion
7	Biallelic *TP53* alteration (Single *TP53* mutation and deletion)	No	∗ Prognostication (monoallelic vs. multiallelic *TP53* alteration)	∗ Chromoanagenesis
8	*MECOM* rearrangement	No	∗ R-IPSS prognostication (*MECOM*)<br>∗ Clinical trial enrollment	
9	*MECOM* (2 different rearrangements)	Yes	∗ R-IPSS prognostication (*MECOM*)<br>∗ Clinical trial enrollment	∗ Cryptic *ETV6* deletion
10	Absence of *TP53* alteration [add(17p) by karyotyping] with a single *TP53* mutation	No	∗ Prognostication (monoallelic vs. multiallelic *TP53* alteration)	

11	*KMT2A* partial tandem duplication	Yes	∗ Prognostication (R-IPSS-M)	
12	*KMT2A* partial tandem duplication	No	∗ Prognostication (R-IPSS-M)	∗ Chromoanagenesis
13	t(3;6) not involving *MECOM* gene	Yes	∗ R-IPSS prognostication (*MECOM*)<br>∗ Clinical trial enrollment	

*Detection essential for prognosis or therapeutic decision making (when suspected or even when apparent by karyotype, this generally requires an additional assay for confirmation)
**Other aberrations, clinical significance not established

Supplementary Figures

Supplementary Figure S1

Schematic overview of the workflow describing the experiment and analysis for optical genome mapping (OGM). Ultra-high-molecular-weight-DNA was extracted from fresh/frozen BM cells, followed by direct label and stain (DLS) labeling, linearization, and sequential imaging. The imaging data was converted to molecules that were assembled de novo to generate consensus genome maps using a reference.

Supplementary Figure S2

Representative images of all types of structural variants noted by optical genome mapping in MDS. (A) Circos plot demonstrating a derivative chromosome, composed of segmental copy number losses in chromosomes 1 and 7, gain in chromosome 1 and t(1;7). (B-G). The data, extracted computationally, can also be interpreted by visualizing the changes in sequence patterns between the sample (genome map of the sample of interest) compared to reference (green). Compared to the reference, the alterations include deletion (B), insertion ©, tandem duplication (D), inversion (E), inter-chromosomal translocations in cis (F) and trans (G). Additional alterations seen on circos plot include a three-way translocation (H), chromoanagenesis (I) and copy-neutral loss-of-heterozygosity in chromosome 5 (J).

Supplementary Figure S3

Additional value of OGM in MDS with complex karyotype settings. A-B. Representative chromosomes shown on circos plots demonstrated a higher genomic complexity than apparent by traditional karyotype (listed below each of the circus plots). C. Chromoanagenesis involving chromosomes 1 and 11, was evident by karyotype. D. OGM resolved the “additional material of unknown significance” and marker in the karyotype (listed below) by demonstrating t(2;11) in addition to other aberrations.

Supplementary Figure S4

Distribution of cryptic SVs detected by optical genome mapping (OGM) in different clinico-biological MDS subsets. The distribution of clinically significant SVs detected only by OGM but cryptic by conventional chromosome banding analysis (CBA) across different CCSS risk groups (A), R-IPSS categories (B), based on number of gene mutations (C) and mutational/cytogenetic subtypes (D) (shown in pink color).

SUPPLEMENTAL FIGURE 1

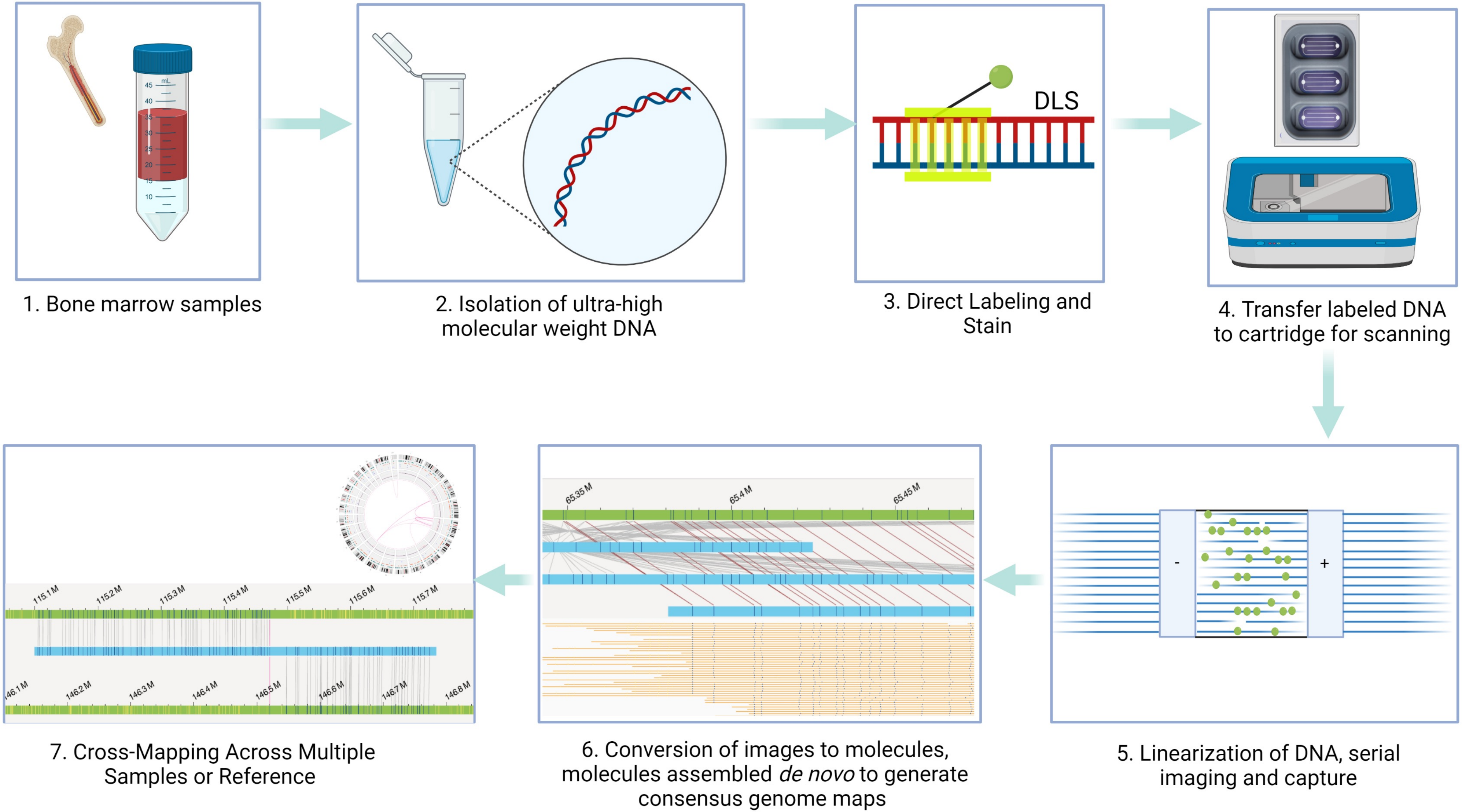

SUPPLEMENTAL FIGURE 2

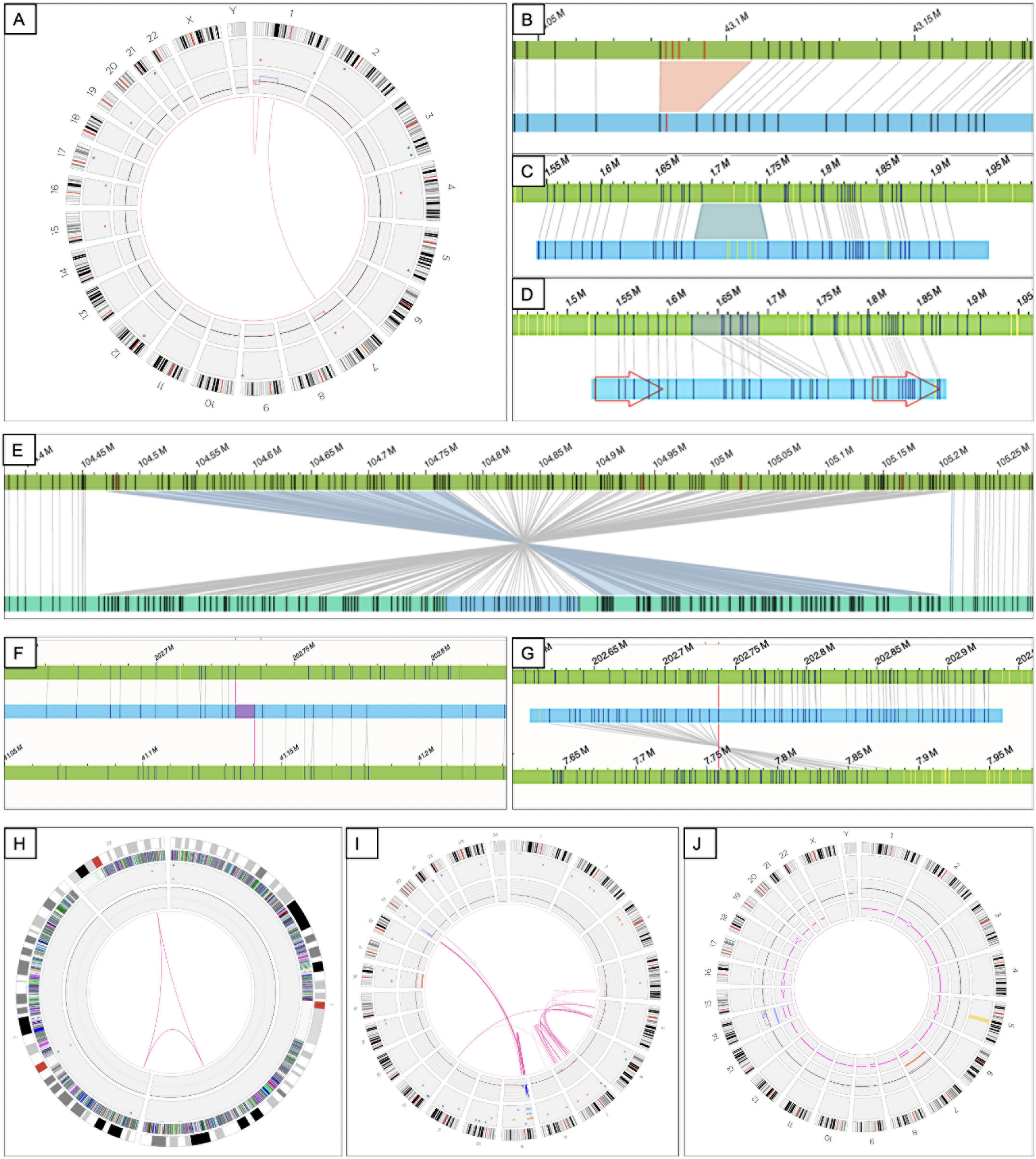

Supplementary Figure 3

A

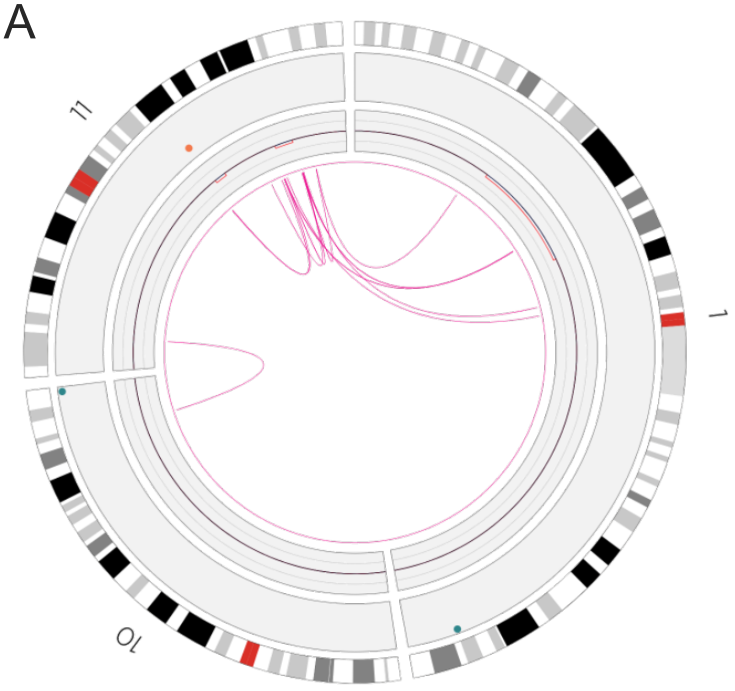

46,XY,add(1)(p22)der(11)t(1;11)(p34;q22)

B

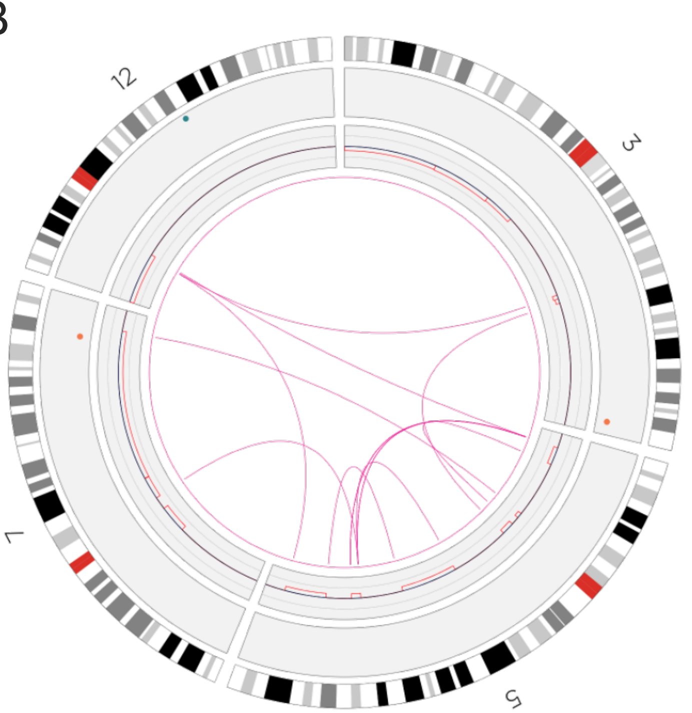

45,XY,der(3;7)(q10;q10)t(3;7)(q12;q21),del(3)(q21),
add(5)(q11.2),del(12)(p11.2)

C

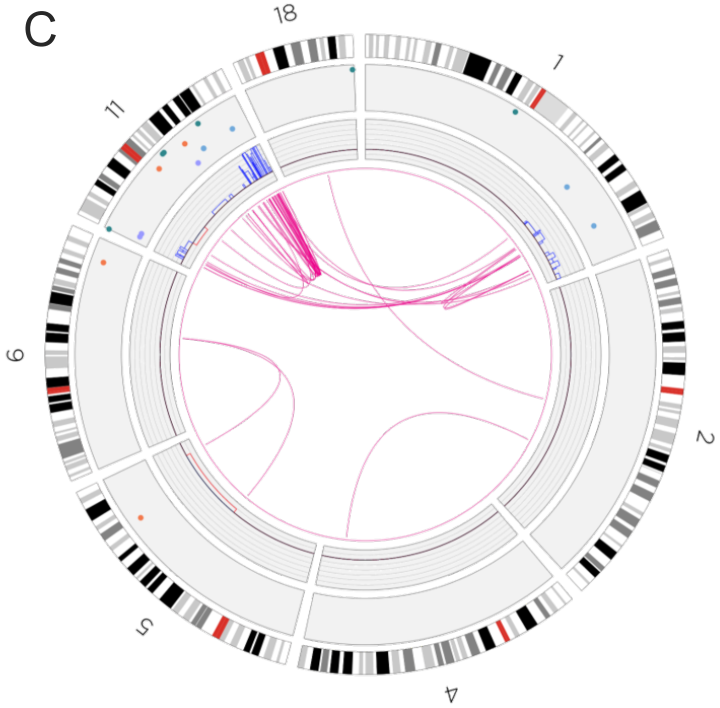

Chromoanagenesis

D

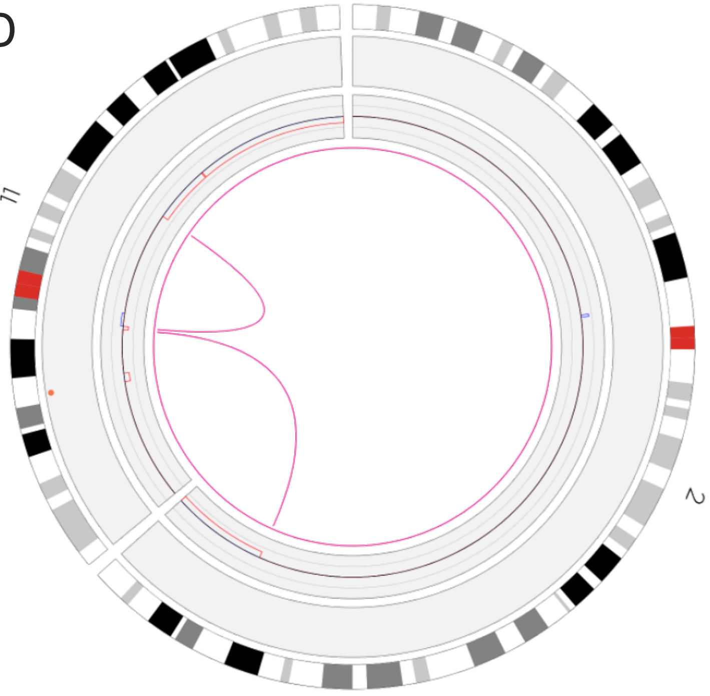

45~47,XY,add(2)(q33),-11,del(11)(q14q23),+1~2mar

SUPPLEMENTAL FIGURE 4

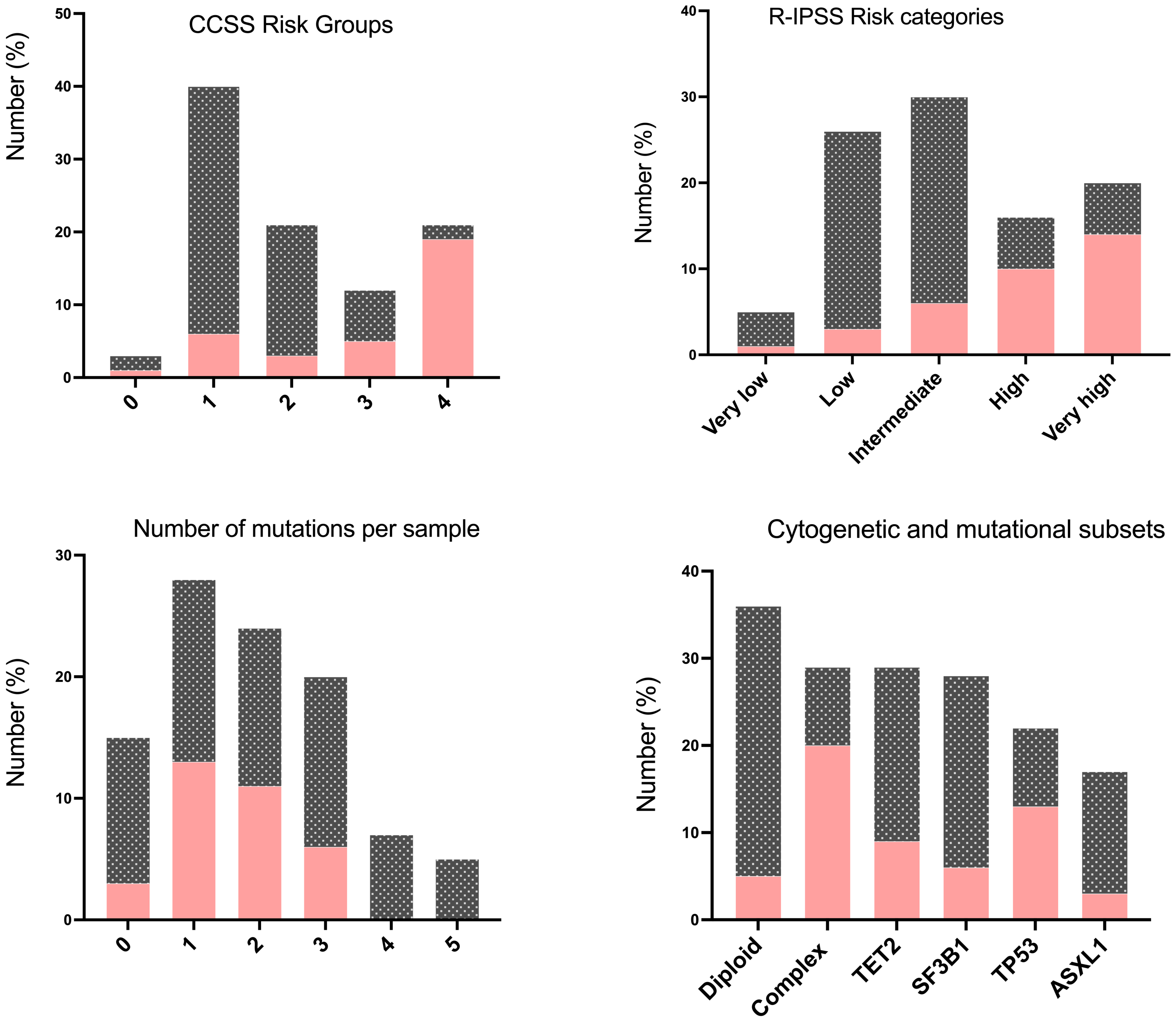

Supplement: Supplementary file 1 — Supplementary Material [file 41375_2022_1652_MOESM1_ESM.pdf]
